# Primary lymphoma of the prostate treated with rituximab-based chemotherapy: a case report and review of the literature

**DOI:** 10.4076/1757-1626-2-8875

**Published:** 2009-08-11

**Authors:** Amina Taleb, Nabil Ismaili, Rhizlane Belbaraka, Abdellah Bensouda, Ibrahim Elghissassi, Omar Elmesbahi, Jean Pierre Droz, Hassan Errihani

**Affiliations:** 1 Department of Medical Oncology, National Institute of Oncology, Rabat, Morocco; 2 Department of Medical Oncology, Centre Léon-Bérard, 28 Rue Laennec, Lyon-69008, France; 3 Department of Urology, IBN-SINA Hospital, Rabat, Morocco; 4 Department of Medical Oncology, Hassan II Hospital, Fes, Morocco

## Abstract

**Introduction:**

Primary lymphoma of the prostate is very rare. In this paper we present a case of early stage non-Hodgkin lymphoma of the prostate managed with six cycles of rituximab-based chemotherapy, and review the related literature.

**Case presentation:**

An 84-year-old man was admitted to our hospital having signs and symptoms suggestive of prostatic disease for 3 years. Histological and immunocytochemical studies of trans-urethral biopsy of the prostate showed diffuse large B-cell lymphoma. Radiological assessment of disease confirmed the diagnosis of early stage lymphoma of the prostate. The patient was managed by 6 of rituximab 375 mg/m^2 ^on day 1, cyclophosphamide 750 mg/m^2 ^on day 1, doxorubicin 50 mg/m^2 ^on day 1, vincristine 1.4 mg/m^2 ^on day 1, and prednisone 50 mg/m^2 ^on days 1 to 5 with complete clinical and radiological response. He remained disease free, until now, 30 months after the end of chemotherapy.

**Conclusion:**

According to the literature, the treatment and prognosis of primary lymphoma of the prostate is the same as that of other nodal lymphomas. Rituximab-based regimen should be considered in the management of prostatic diffuse large B-cell lymphoma.

## Introduction

Primary lymphoma of the prostate is extremely rare representing approximately 0.2 to 0.8% of extra nodal lymphoma and 0.1% of all prostate neoplasms [1-3]. In this paper, we present a case of prostatic early large B-cell lymphoma of an 84-year-old man managed successfully with rituximab-based chemotherapy and review of the related literature.

## Case presentation

An 84-year-old Moroccan Caucasian man admitted in our institution with difficulty of urination, suggestive of urinary obstruction for 3 years and having systemic symptoms (B symptoms) (Fever, shudder, profuse sweat, without weigh loss) for 6 months. He had no relevant past medical history. At first diagnosis, the performance status was equal to 1.0. Digital rectal examination showed firm prostate weighing 30 g. Ultrasound prostate and kidneys examination showed voluminous prostate (35 g) and bilateral hydronephrosis. The serum tumour marker serum Prostate-specific antigen (PSA) was negative, being 2.97 ng/ml (normal <4 ng/ml).

Histological and immunohistochemical studies of needle biopsies of prostatic tissue obtained by transurethral resection showed diffuse large B-cell lymphoma (DLBCL). Most of the neoplastic cells were positive for Cluster of differentiation-20 (CD-20) and leukocyte common antigen (LCA).

**Figure 1:**
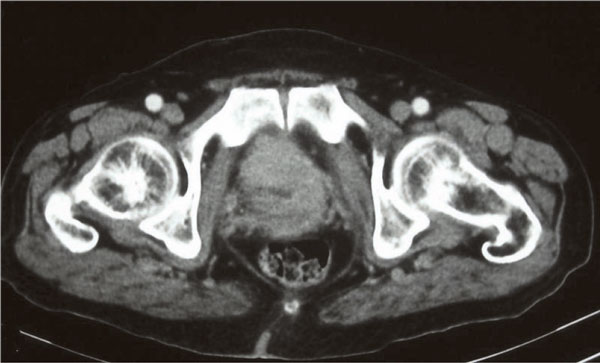
**CT scan of the pelvis shows the tumoral process infiltrated the base of the prostate gland**.

**Figure 2:**
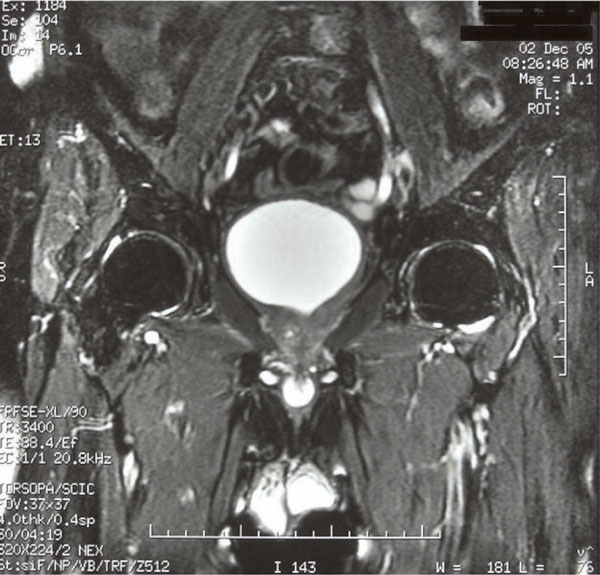
**T2 weighted coronal section fat-suppressed shows the process in the left base of the prostate which invades the bladder base**.

**Figure 3:**
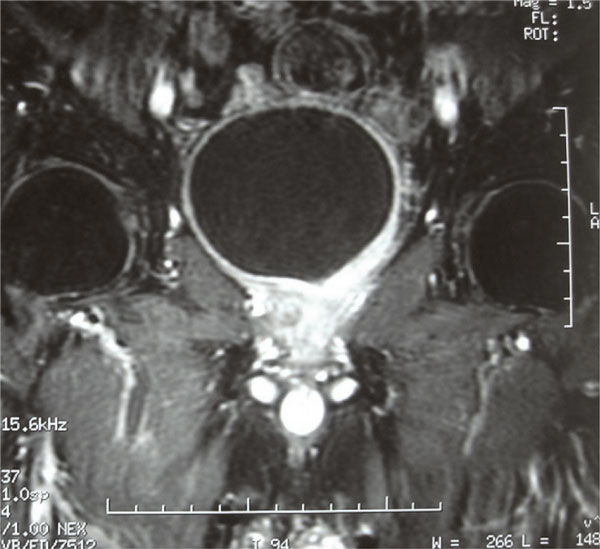
**T1 weighted coronal section after injection of gadolinium shows abnormal enhancement of the prostate and the bladder base**.

**Figure 4:**
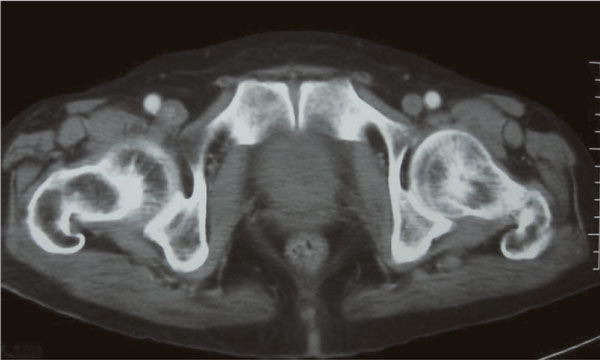
**CT scan of the pelvis performed after chemotherapy shows complete radiological response of the prostatic tumour**.

Computed tomography (CT) scan and magnetic resonance imaging (MRI) of the pelvis showed a large prostatic tumour (Figure 1). The tumour invaded the base of the bladder (Figure 2 and Figure 3). No evidence of distant metastasis on lung and abdomen was shown by CT scan. The erythrocyte sedimentation rate was increased, being 65 mm in the first hour. A bone marrow biopsy showed no abnormalities. The patient was staged IEBb according to the Ann Arbor classification system. He was managed with six cycles of rituximab 375 mg/m^2 ^on day 1, cyclophosphamide 750 mg/m^2 ^on day 1, doxorubicin 50 mg/m^2 ^on day 1, vincristine 1.4 mg/m^2 ^on day 1, and prednisone 50 mg/m^2 ^on day 1 to 5 (RCHOP regimen). The evaluation was done by abdomino-pelvic and chest CT scans. The first CT scan of the pelvis performed after 3 cycles of chemotherapy showed partial radiological response of the prostatic tumour and the second (Figure 4) performed after the end of the 6 cycles of treatment showed complete radiological response. The patient remained disease free, until now, 30 months after the end of the chemotherapy.

## Discussion

Adenocarcinoma represents more than 90% of all prostatic malignancies. Other histological subtypes of prostatic cancers represent only 5 to 10%. Involvement of the prostate by malignant lymphoma is a well-known late manifestation of advanced nodal disease [4]. However, primary lymphoma of the prostate is extremely rare representing only 0.2 to 0.8% of extra nodal lymphoma and 0.1% of all prostate neoplasms [1-3]. It is estimated that less than 150 cases have been described in the world literature.

The mean age at diagnosis is 62 years [5]. The criteria for the diagnosis of primary prostatic lymphoma were defined by Bostwick *et al*. [5]. Tumours were considered to be primary for patients having the following: (1) symptoms of prostatic enlargement at the beginning of the disease; (2) predominant involvement of prostate; and (3) no involvement of lymph nodes, blood, liver, or spleen [5].

Almost all patients diagnosed with prostatic lymphoma, whether primary or secondary, present symptoms of lower urinary obstruction [2,3,5]. Some patients present pain or hematuria, and others present systemic symptoms. PSA is increased for 20% of alls cases. On digital rectal examination the prostate appears diffusely enlarged or nodular, and firm [6]. For our patient, we showed urinary obstruction, systemic symptoms and firm prostate weighing 30 g on digital rectal examination.

Pathological diagnosis is usually obtained by examination of needle biopsies of prostatic tissue obtained by transurethral resection. Occasionally lymphoma is diagnosed as an incidental finding in a radical prostatectomy specimen removed for known prostatic adenocarcinoma [6]. We may also encounter lymphoma/leukemia as an incidental finding in approximately 0.2 to 1.2% of pelvic lymph node resections performed at radical prostatectomy [7,8].

The DLBCL is the most common type of primary lymphomas of the prostate, but primary prostatic small lymphocytic lymphoma, follicular lymphomas, Burkit lymphomas, MALT lymphomas, and mantle cell lymphomas have also been reported [2,3,5,9].

Because primary lymphoma of the prostate is rare, little is known of its optimal management. In the retrospective review of 62 patient performed by Bostwick *et al. *[5], 47% of patients died of lymphoma, the specific 5-year survival was only 33%. 73% of patients with primary prostatic lymphoma developed extra prostatic disease 1 to 59 months after diagnosis. There were no significant differences in survival between patients receiving different therapies: chemotherapy, chemotherapy and radiotherapy or surgery only. There were no significant differences between patients with primary or secondary prostatic lymphoma, or between patients with different types of lymphoma [5]. However, patients in this retrospective study concern cases found in different centres during 58-year period, and it is unlikely that these data reflect the results that would be achieved using current therapeutic regimens. Indeed, a number of more recent case studies have reported good outcomes for patients with high-grade primary prostatic lymphoma treated with anthracycline-based chemotherapy with or without radiotherapy [2,10,11]. Other cases with localised diffuse large B-cell lymphoma were managed successfully with radiotherapy only [12]. Rituximab in combination with CHOP regimen is considered as the standard treatment for patients with advanced stage DLBCL [13]. Studies of rituximab use in the management of gastrointestinal and other extra-nodal lymphoma are ongoing in research programs. And the first results appear to be encouraging. To our knowledge, the present case is the first case of early primary lymphoma of the prostate which was managed successfully with RCHOP chemotherapy.

Because of the rarity of the disease, the prognosis of primary prostatic lymphoma is not clear. It remains uncertain whether the prognosis of prostatic lymphoma is significantly worse or equivalent to nodal lymphoma. In one review of 23 cases of Japanese primary prostatic lymphoma the authors suggest that patients with this malignancy respond well to chemotherapy and could possibly be cured when the disease is confined to prostatic region [14]. Others authors suggest that prognosis of these malignancies dependent on the histological type and stage of the individual tumour, as it is the case of other non-Hodgkin's lymphomas [12]. In concordance with theses conclusions, the prognosis and treatment of other extra nodal lymphoma are the same as that of nodal lymphomas [13,15].

## Conclusion

Primary lymphoma of the prostate is rare. In most cases the diagnosis was induced by urinary obstruction. Because of the rarity of disease, there is no standard treatment, universally accepted of primary DLBCL of the prostate. For early stage, the combination of chemotherapy and radiotherapy appears to be a logical option. For advanced disease, rituximab in association with CHOP chemotherapy should be considered as the first-choice treatment.

## Abbreviations

CD-20: cluster of differentiation-20; CT: computed tomography; DLBCL: diffuse large B-cell lymphoma; LCA: leukocyte common antigen; MALT: mucosal-associated lymphoid tissue; MRI: magnetic resonance imaging; PSA: serum prostate-specific antigen.

## Competing interests

The authors declare that they have no competing interests.

## Consent

Written informed consent was obtained from the patient for publication of this case report and accompanying images. A copy of the written consent is available for review by the Editor-in-Chief of this journal.

## Authors' contributions

NI and AT contributed equally to this work. All authors made significant contributions by making the diagnosis, intellectual input in the case and writing the manuscript.
